# Cerebral venous sinus thrombosis secondary to neurocryptococcosis

**DOI:** 10.1590/0037-8682-0143-2023

**Published:** 2023-07-24

**Authors:** Luiz Fernando Monte Borella, Mariângela Ribeiro Resende, Fabiano Reis

**Affiliations:** 1 Universidade Estadual de Campinas, Departamento de Radiologia, Campinas, SP, Brasil. Universidade Estadual de Campinas Departamento de Radiologia Campinas SP Brasil; 2 Universidade Estadual de Campinas, Departamento de Clínica Médica, Campinas, SP, Brasil. Universidade Estadual de Campinas Departamento de Clínica Médica Campinas SP Brasil

**FIGURE 1:**
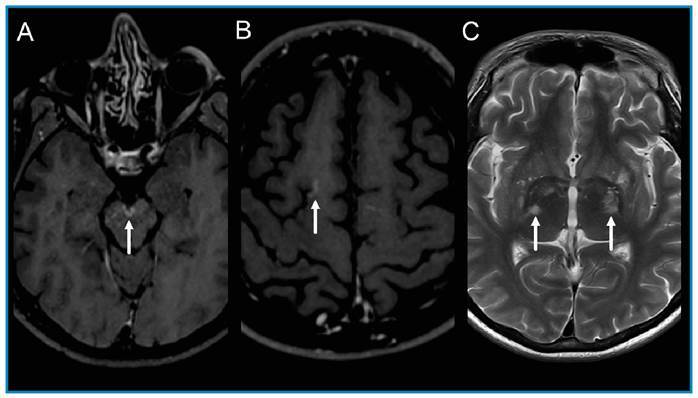
Post-contrast axial T1-weighted images showing leptomeningeal enhancement of the interpeduncular fossa and right superior frontal sulcus (arrows, A and B). Axial T2-weighted image showing dilated perivascular spaces in the basal ganglia (arrows, C).

**FIGURE 2:**
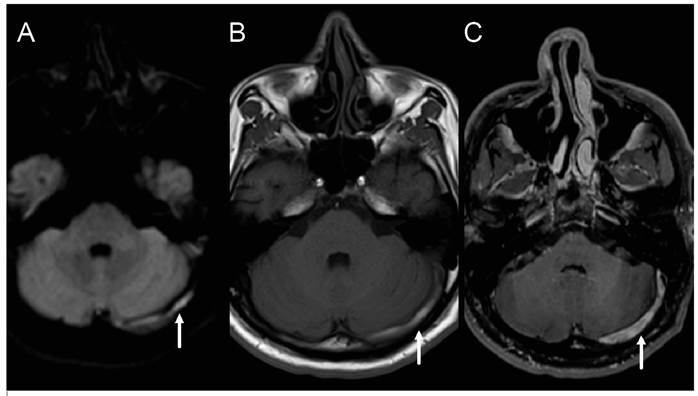
Axial diffusion-weighted images (A), T1-weighted images before contrast administration (B) and post-contrast T1-weighted images (C) showing partial thrombosis of the left transverse sinus (arrows).

A 41-year-old man presented with headache, fever, mild bilateral papilledema, and neck stiffness. Brain magnetic resonance imaging (MRI) showed leptomeningeal enhancement and dilated perivascular spaces with peripheral enhancement in the basal ganglia (Figure 1), and cerebral venous thrombosis (Figure 2). Cerebrospinal fluid (CSF) examination revealed 22 white blood cells (94% lymphocytes), with glucose and protein concentrations of 28 mg/dL and 137 mg/dL, respectively. CSF culture was positive for *Cryptococcus neoformans.* A test for human immunodeficiency virus was positive (viral load: 537,969 copies/mL) and CD4+ count was 11 cells/mL. Treatment was started with the induction phase with amphotericin B lipid complex (400 mg, once daily) and fluconazole (600 mg, twice daily) for 30 days. Symptom regression was observed, and the consolidation phase was maintained for eight weeks with fluconazole (450 mg twice a day).

Central nervous system cryptococcosis produces a variety of MRI features^1^. Leptomeningeal enhancement and the occurrence of an inflammatory reaction are minimal, and result in the production of mucoid material within the subarachnoid space, a process that may extend to the perivascular spaces^1^.

Cerebral venous sinus thrombosis is a rare complication of cryptococcal meningitis and is usually related to co-infection with HIV. The clinical presentation of cerebral venous thrombosis in the setting of neuroinfection is variable, with a combination of brain MRI and MRI venography being the most sensitive imaging approach for diagnosis^2^. When there is concomitant HIV infection, the occurrence of a low CD4+:CD8+ ratio, high viral count, and lack of antiretroviral therapy are risk factors for procoagulant alterations disturbances^3^.
